# Decompressive craniectomy to cranioplasty: a retrospective observational study using Hospital Episode Statistics in England

**DOI:** 10.1136/bmjsit-2023-000253

**Published:** 2024-06-03

**Authors:** Harry Mee, J M Harris, T Korhonen, F Anwar, A J Wahba, Michael Martin, G Whiting, E Viaroli, I Timofeev, A Helmy, Angelos G Kolias, Peter J Hutchinson

**Affiliations:** 1 Clinical Neurosciences, University of Cambridge, Cambridge, UK; 2 University of Bristol, Bristol, UK; 3 Department of Clinical Neurosciences, Cambridge University Hospitals NHS Foundation Trust, Cambridge, UK; 4 Neurosurgery, University of Oulu, Oulu, Finland; 5 University of Leeds, Leeds, UK; 6 Obex Technologies Ltd, Cambridge, UK

**Keywords:** Cohort Study

## Abstract

**Objectives:**

To investigate the longitudinal trends of decompressive craniectomy (DC) following traumatic brain injury (TBI) or stroke and explore whether the timing of cranial reconstruction affected revision or removal rates using Hospital Episode Statistics (HES) between 2014 and 2019.

**Design:**

Retrospective observational cohort study using HES. The time frame definitions mirror those often used in clinical practice.

**Setting:**

HES data from neurosurgical centres in England.

**Participants:**

HES data related to decompressive craniectomy procedures and cranioplasty following TBI or stroke between 2014 and 2019.

**Main outcome measures:**

The primary outcome was the timing and rate of revision/removal compared with cranioplasty within <12 weeks to ≥12 weeks.

**Results:**

There were 4627 DC procedures, of which 1847 (40%) were due to head injury, 1116 (24%) were due to stroke, 728 (16%) were due to other cerebrovascular diagnoses, 317 (7%) had mixed diagnosis and 619 (13%) had no pre-specified diagnoses. The number of DC procedures performed per year ranged from 876 in 2014–2015 to 967 in 2018–2019. There were 4466 cranioplasty procedures, with 309 (7%) revisions and/or removals during the first postoperative year. There was a 33% increase in the overall number of cranioplasty procedures performed within 12 weeks, and there were 1823 patients who underwent both craniectomy and cranioplasty during the study period, with 1436 (79%) having a cranioplasty within 1 year. However, relating to the timing of cranial reconstruction, there was no evidence of any difference in the rate of revision or removal surgery in the early timing group (6.5%) compared with standard care (7.9%) (adjusted HR 0.93, 95% CIs 0.61 to 1.43; p=0.75).

**Conclusions:**

Overall number of craniectomies and the subsequent requirements for cranioplasty increased steadily during the study period. However, relating to the timing of cranial reconstruction, there was no evidence of an overall difference in the rate of revision or removal surgery in the early timing group.

**WHAT IS ALREADY KNOWN ON THIS TOPIC**

- Clinical trials over the past 10 years have demonstrated the efficacy of decompressive craniectomy to aid in the management of traumatic brain injury, with its utilisation also increasing in the stroke population, but there is very little data exploring the impact in clinical practise. Cranial reconstruction follows, often many months later, but the optimal time for the procedure is not known.

**WHAT THIS STUDY ADDS**

- This study offers an overview of the total number of decompressive craniectomy and cranioplasty procedures in England and provides evidence to aid in the discussion of the optimal timing of cranioplasty.

**HOW THIS STUDY MIGHT AFFECT RESEARCH, PRACTICE OR POLICY**

- Understanding the current clinical landscape with regard to procedure numbers and timing may help in the development of trauma and stroke pathways, especially in terms of rehabilitation services, provision and outcome.

## Introduction

Decompressive craniectomy (DC) is a neurosurgical procedure aimed at managing raised intracranial pressure that is refractory to maximal medical management in the context of traumatic brain injury (TBI) or stroke. Skull repair with cranioplasty often occurs many months later, intending to restore a degree of mechanical protection to the brain and potentially improve neurological function.^1^ Cranial reconstruction is an important component in rehabilitating patients following brain injury and can significantly impact the trajectory of recovery.

Historically, cranioplasty was undertaken beyond 6 months following DC because it was thought that an earlier cranioplasty increases the risk of infection.^2–4^ However, recent systematic reviews have challenged this, and a growing body of evidence supports an association between earlier cranioplasty with improved outcomes.^1 5–7^ As a result, the timing of cranioplasty is a topic of continued interest and debate, with the delineation classically reported as within 12 weeks or after 12 weeks^6^ from DC. Although timing categories can appear somewhat artificial, as the procedure should be performed when clinically most appropriate, the consensus meeting on post-traumatic cranioplasty in 2018 highlighted the importance of defining thresholds for clinical benchmarking and research purposes, with an 87% agreement reached regarding the following time frames: ultra-early, up to 6 weeks; early, 6 weeks to 3 months; intermediate, 3–6 months and delayed, more than 6 months.^8^

Independent of timing, cranial reconstruction is often beneficial for the patient, but does have a significant risk profile, with overall complications reported at approximately 20%.^6^ In addition, this surgical pathway commonly occurs following severe brain injury, and these patients often require complex disability management and structured inpatient rehabilitation. The UK Cranial Reconstruction Registry^9 10^ provides nationally collated figures on operative details of cranioplasty insertions and related repeat procedures, but there is currently limited data on national practice patterns and associations with the timing of cranioplasty. Understanding such factors would be helpful for both clinical care and benchmarking as well as for service evaluation and rehabilitation pathway planning.

This observational study aimed to investigate the longitudinal trends of DC following TBI or stroke and explore the timing of cranial reconstruction and whether these impacted subsequent revision or removal using UK Hospital Episode Statistics (HES) data between 01 April 2013 and 31 March 2019.

## Methods

### Defining study population

HES is an administrative database of National Health Service hospitals in England. Although its primary function is to aid hospital trusts’ financial reimbursements, it may also be used for clinical research and audit.^11^ HES data include the Office of Population Censuses and Surveys (OPCS) classification of interventions and procedures data set (current version 4.9 (April 2020)), allowing for the identification of relevant surgical procedures during hospital admissions. The OPCS codes used for cranioplasty (V01.1–9) have remained the same since 1987; DC procedures were only identified by a unique code (V03.7) since March 2014. Please see the online supplemental appendix for the OPCS codes used in this study.

10.1136/bmjsit-2023-000253.supp1 Supplementary data

#### Diagnoses

The International Statistical Classification of Diseases and Related Health Problems (ICD) is a medical classification list by the WHO that codes for diseases, signs and symptoms, abnormal findings, issues, social circumstances and external causes of injury or diseases, which were used to identify the primary diagnoses of DC and cranioplasty patients in the present study. The International Classification of Diseases 10th edition (ICD-10) was used for disease classification in this study. The study population defined by the OPCS codes was divided into three categories based on the primary reason for decompression per the ICD-10 classification: head injury, stroke and cerebrovascular event (CVE) (excluding stroke) (eg, subarachnoid haemorrhage) (online supplemental appendix 1).

### Identification of patients

Craniectomy and cranioplasty procedures were independently identified and linked where possible.

#### Identification of craniectomy and cranioplasty procedures

Patients aged 16 years or older were included in this study. Admitted patient care (APC) data between 01 April 2014 and 31 March 2020 were analysed in relation to OPCS procedure codes in any position for decompressive craniectomy (V03.7) or related to the opening of the cranium (V03.7–9 and/or V05.8) and cranium repair (V01.1–9). In addition, ICD-10 diagnosis codes related to haematoma evacuation (A40.1/A41.1) were analysed (table 1). All identified patients who were linked to admissions between 01 April 2013 and 31 March 2020 were queried from the HES database (table 1) alongside demographic data.

**Table 1:** Demographics of 1823 patients who underwent decompressive craniectomy and linked cranioplasty

	Stroke	CVE (not stroke)	Head injury	Mixed diagnosis	Total
Total numbers	**353 (19%)**	**248 (14%)**	**949 (52%)**	**273 (15%)**	**1823**
Age craniectomy: median (IQR)	49 (41–56)	50 (40–59)	37 (26–52)	47 (33–60)	
Age at craniectomy; n (%)					
16–35	54 (9)	45 (7)	429 (71)	78 (13)	606
36–50	141 (25)	83 (15)	260 (47)	73 (13)	557
51–65	145 (29)	87 (17)	194 (39)	77 (15)	503
66–80	13 (9)	29 (21)	60 (43)	39 (28)	141
81+	0	4	6	6	16
Sex; n (%)					
Male	206	113	744	180	1243 (68)
Female	147	135	205	93	580 (32)
Comorbidities					
Diabetes	38	14	49	24	125
Hypertension	115	97	129	64	405
AF	36	12	34	12	94
TIA	10	15	2	2	29
Stroke	1	0	1	0	2
IHD	26	20	32	11	89
Length of stay for craniectomy admission; median (IQR) days	114 (69–172)	89 (31–166)	67 (35–143)	47 (14–107)	

AF, atrial fibrillation; CVE, cerebrovascular event; IHD, ischaemic heart disease; TIA, transient ischaemic attack.

Clinically, there are surgical cases in which the decision to undertake decompressive craniectomy occurs intraoperatively, and this can lead to difficulties with coding, with the DC code (V03.7) often omitted. To mitigate this, the OPCS code for haematoma evacuation (A40.1 or A41.1) was reviewed, and if there was no coded DC (V03.7) at any time point but a linked cranium repair (V01.1–9), which was performed through cranioplasty, an assumption was made that the patient underwent a craniectomy (V03.7).

### Statistical analysis

#### Analysis 1: total numbers

**Figure 1:**
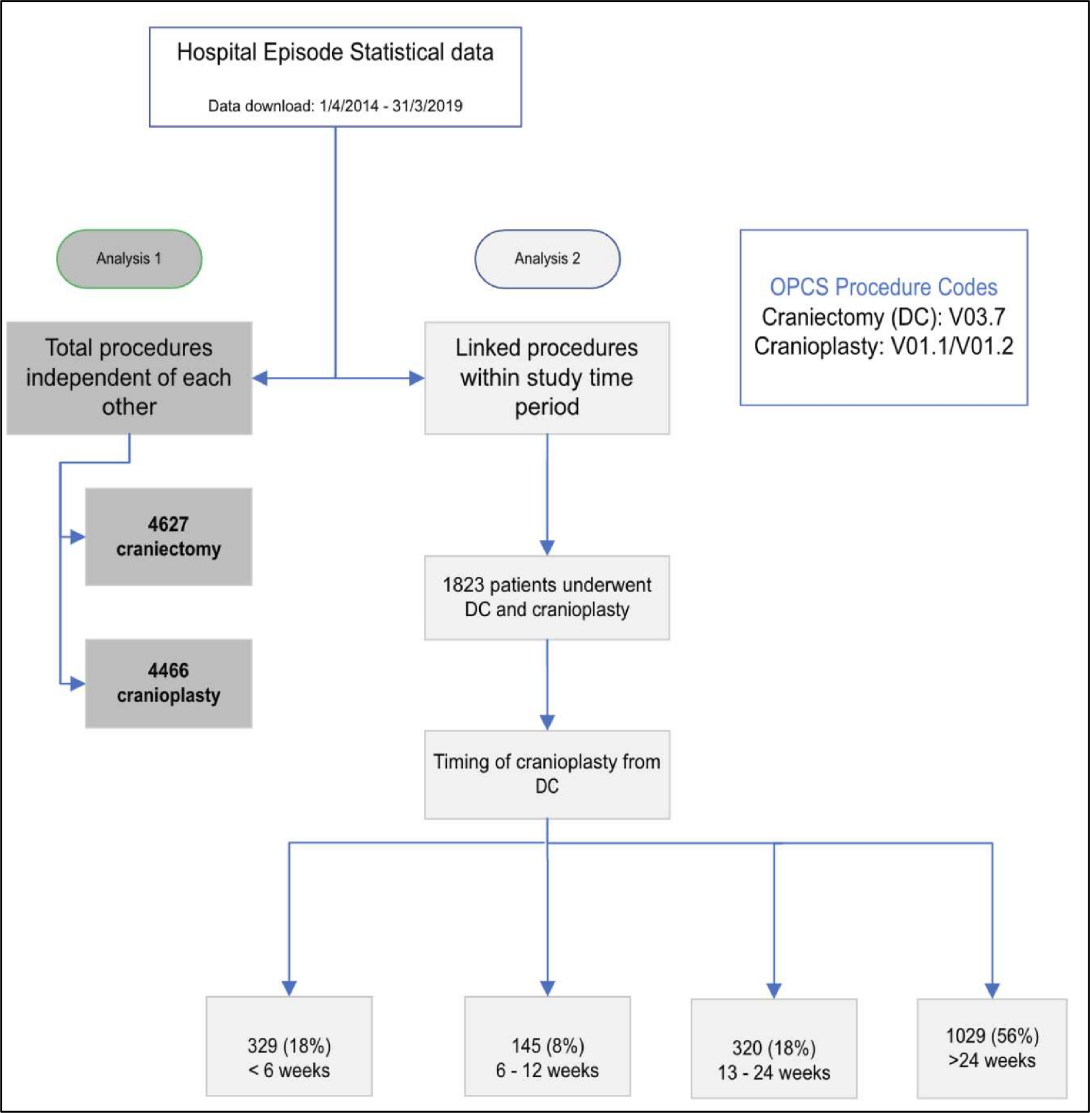
The study flowchart describing the workflow of the two study analyses. DC, decompressive craniectomy; OPCS, Office of Population Censuses and Surveys.

Descriptive statistics were used to understand all data between 01 April 2014 and 31 March 2019, describing the total numbers of DC and cranioplasty procedures independently of each other (figure 1). In addition, annual numbers of DC and mortality at index admission were calculated, as well as the total number of cranioplasty procedures (not necessarily linked to a DC during the study period) with removal/revision rates also reported.

#### Analysis 2: craniectomy with linked cranioplasty

Analysis 2 included only patients who underwent craniectomy and cranioplasty during the study period.

Descriptive statistics were used to summarise the basic demographic information, including age, sex and comorbidities. Number of procedures in relation to the timing of cranioplasty from craniectomy were analysed: first, before versus after 12 weeks. Further subcategorisation into less than 6 weeks, 6–12 weeks, 13–24 weeks and >24 weeks was then undertaken as a subgroup analysis. The Charlson Comorbidity Index (CCI) was calculated using the HES admission data from the year before craniectomy, using the methods described by Maringe *et al*.^12^

The primary outcome was the timing and rate of revision/removal comparing cranioplasty within <12 weeks to after ≥12 weeks (occurrence of HES codes V01.4–5; table 1) and was undertaken using Cox’s proportional hazards regression. The model was adjusted for factors considered as potential confounders a priori: age, sex, timing, material type, primary diagnosis and CCI score. Due to the potential clustering of sites, the hospital provider at the time of cranioplasty was included in the model as a frailty term. Patients were censored at their date of death if this was known; this was only the case for in-hospital mortality.

All analyses were performed using Stata V.17 (StataCorp LP; College Station, Texas, USA).

## Results

### Analysis 1: total numbers

#### Craniectomy

A total of 4627 coded DC procedures were performed during the study period. Of these, 1847 (40%) patients had a head injury diagnosis, 1116 (24%) had a stroke diagnosis and 728 (16%) had a CVE diagnosis as defined by the ICD-10 classification. In addition, 317 (7%) patients had a mixed diagnosis and 619 (13%) had no prespecified diagnoses. A total of 1132 (25%) patients died during the same admission in which DC occurred and 123 (3%) died during later admission. A total of 2804 (61%) DC procedures had no later cranioplasty coded during the study period.

The number of DC procedures categorised by primary diagnosis increased by 12% from 876 on April 14 to March 15 to 967 on April 18 to March 19. There was a 42% (n=183–260) increase in the stroke cohort and a 14% (n=339–385) increase in the head injury cohort.

#### Cranioplasty

A total of 4466 cranioplasty procedures were performed. A total of 309 (7%) cranioplasty procedures required revision or removal during the first postoperative year.

### Analysis 2: craniectomy with linked cranioplasty

#### Craniectomy

A total of 1823 patients underwent DC and subsequent cranioplasty during the study period, of which 1243 (68%) were men. A total of 1550 patients had a single-coded diagnosis leading to DC: 949 (52.1%) head injury, 353 (19.4%) stroke, 248 (13.6%) CVE and 273 (15.0%) mixed diagnoses.

The median age at the time of craniectomy was 50 years for CVE, 49 for strokes and 37 for head injuries (table 2). The median length of index hospital admission was longest in the stroke cohort (114 days) and shortest in the head injury cohort (67 days) (table 1).

**Table 2:** Timing of cranioplasty procedures from DC by cranioplasty consensus meeting adopted time frames

	Stroke	CVE	Head injury	Mixed diagnosis	Total
Total (%)	**353 (19)**	**248 (14)**	**949 (52)**	**273 (15)**	**1823**
Time to CP					
<6 weeks	21 (6)	48 (19)	172 (18)	88 (32)	329 (18)
6 to <12 weeks	16 (4)	16 (6)	97 (10)	16 (6)	145 (8)
12 to <24 weeks	51 (14)	34 (14)	198 (21)	37 (14)	320 (18)
≥24 weeks	265 (75)	150 (60)	482 (51)	132 (48)	1029 (56)

CP, cranioplasty procedure; CVE, cerebrovascular event; DC, decompressive craniectomy.

#### Cranioplasty

Of the 1823 cranioplasty procedures, 1550 (85%) used synthetic materials, and 273 (15%) used autologous. There were 138 (8%) cranioplasty revisions/removals, of which 69 (50%) were performed on the same admission as the primary cranioplasty insertion.

#### Timing of cranioplasty

**Figure 2:**
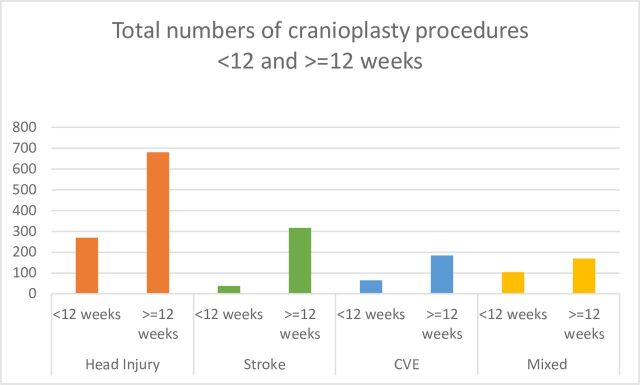
Total numbers of cranioplasty procedures categorised by diagnosis and time frame. CVE, cerebrovascular event.

The number of days to cranioplasty insertion from DC ranged from 0 to 1607 (4.4 years), with 387 (21%) occurring after 1 year. The median time to cranioplasty insertion was 195 days (IQR 79–335 days). Of the patients, 1349 (74%) underwent cranioplasty 12 weeks or more after DC. The head injury group had the highest number, 269/949 (28%) within 12 weeks (figure 2).

There was a 28% increase when comparing the numbers of cranioplasty procedures performed within 12 weeks between April 14 to March 15 (n=67) and April 18 to March 19 (n=86), with 576 (32%) cranioplasty insertions that occurred during the same admission as the DC.

#### Readmissions in the first 12 months from cranioplasty insertion

Within 12 months after cranioplasty insertion, 47% of patients had a readmission (median number of admissions for these patients was 2; IQR 1–3; range 1–44). The three most common specialised services that patients were readmitted under were general internal medicine (N=500; 27%), neurosurgical services (N=399; 22%) and emergency medicine services (N=209; 11%). There were 56/1823 (3%) patients with admissions coded as A12.4, the creation of a ventriculoperitoneal shunt.

### Survival analysis

**Figure 3:**
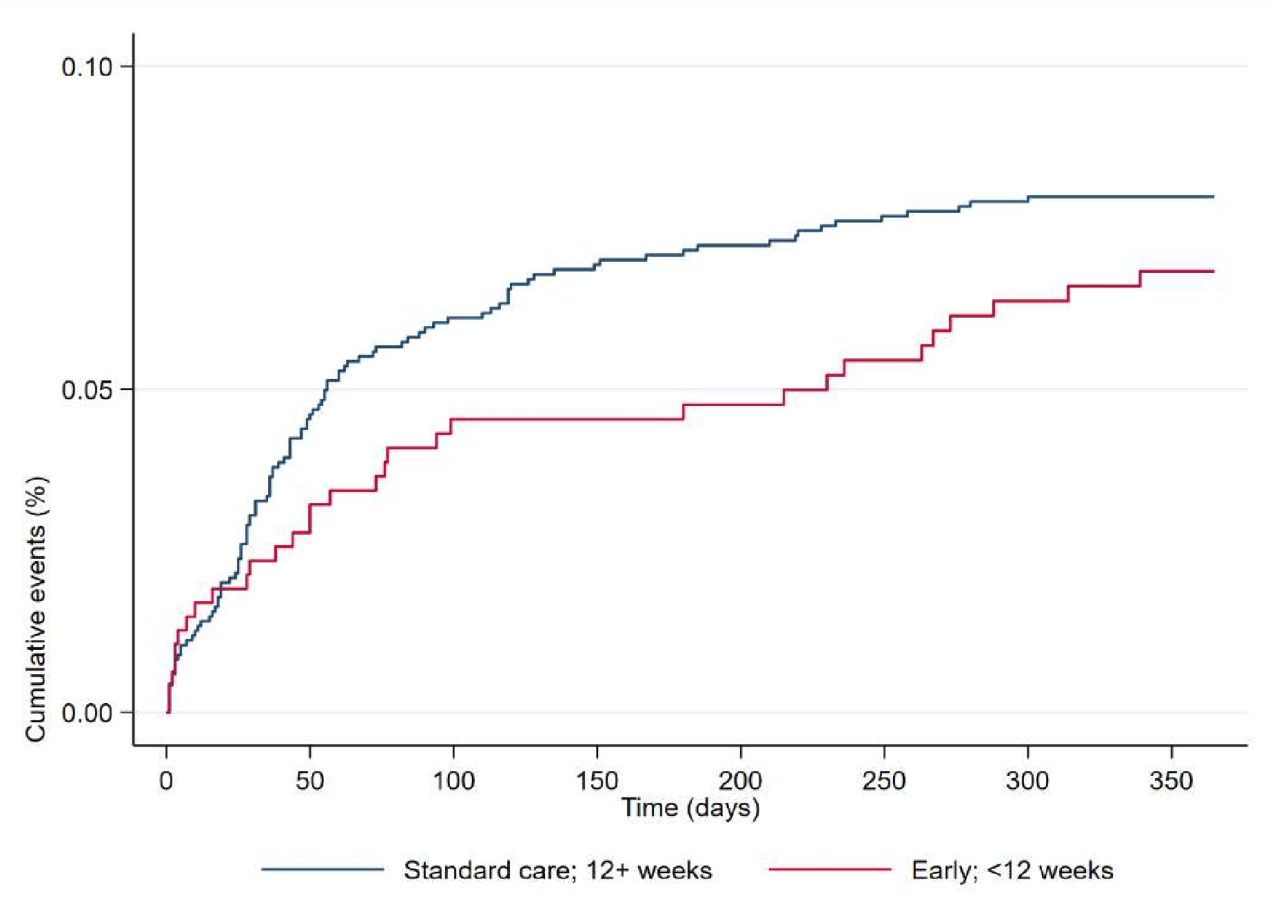
Survival curve of the cranioplasty procedures, depicting the time until the first revision or removal surgery (V01.4 or V01.5, respectively) during the first post-craniectomy year. Patients were censored at their date of death if they were inpatients; otherwise, this date was unknown.

**Figure 4:**
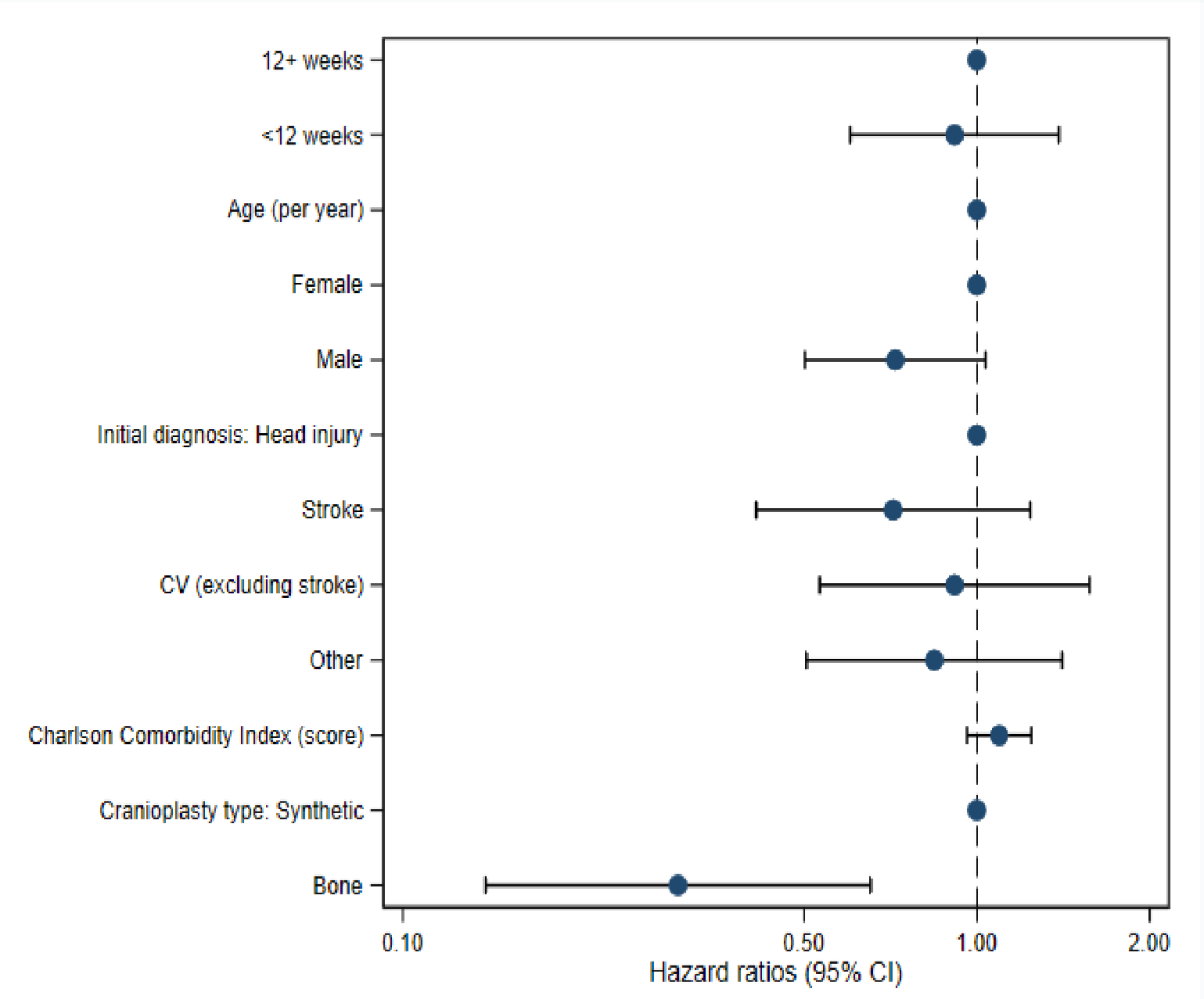
A forest plot of the factors affecting cranioplasty 1-year survival defined as time to revision or implant removal surgery (V01.4 or V01.5, respectively). CV, cerebrovascular.

There was no significant difference overall, following adjustment, in the rate of revision or removal surgery in the early timing group (6.5%) compared with standard care (7.9%) (HR 0.93, 95% CI 0.61 to 1.43; p=0.75) (figure 3). The relationship between all the factors and revision or removal surgery is shown in figure 4.

## Discussion

This observational study aimed to investigate the longitudinal trends of DC following TBI or stroke and explore the timing of cranial reconstruction and whether these affected the revision or removal rates using HES data between 2014 and 2019.

### Decompressive craniectomy numbers

The number of DC procedures performed per year ranged from 876 in 2014–2015 to 967 in 2018–2019 (April–March), with the greatest change from 183 to 260 per year in the stroke cohort. This could be explained, in part, by randomised trials^13–15^ that showed the benefit of DC in the outcome of patients with middle cerebral artery syndrome. Since the National Institute for Health and Care Excellence (NICE) guidelines from 2008^16^ also reflect this, there has been a broadening in clinical management for the criteria of DC following stroke to include not just middle cerebral artery syndrome but other types of stroke, which has also been reflected in the updated NICE guidelines (NG128).^17^ However, minimal level I evidence or guidelines exist for using DC in CVE diagnoses, such as aneurysmal subarachnoid haemorrhage, although a randomised prospective trial protocol has been published.^18^ The relative lack of evidence compared with ischaemic stroke may be related to the less pronounced utilisation of DC for these indications.

There were 1847 (40%) DC procedures performed due to head injuries. These numbers increased by 14% during the study period, and although this study could not differentiate between primary and secondary DC procedures, this trend could be explained by recent level 1 evidence supporting DC following head injury.^19^

#### Cranioplasty timing

The optimal timing of cranioplasty and its relationship with postoperative complications and neurological recovery remains unknown. There is wide variation in practice patterns nationally and internationally, and delineation of cranioplasty time frames has classically come before and after 12 weeks, but this is continuously challenged. The median time to cranioplasty was 195 days, which was slightly reduced from 244 days, as reported by the UK Cranial Reconstruction Registry^10^ in 2021. In this study, 387 (21%) insertions occurred after 1 year, with the longest being reported at 4.4 years. This reduction in median time to cranial implantation may, in part, reflect current clinical practice since infection rates have been shown to remain constant independent of timing,^6^ meaning clinicians may have greater confidence in conducting cranioplasty earlier.

Patients who were diagnosed with head injury had the highest number of cranioplasty procedures performed <12 weeks, a total of 28% (n=269), compared with only 10% (n=37) of patients who had a stroke. This may be partly due to resource allocation and clinical pathways, with patients who had a stroke often being transferred to neurosurgical centres for surgical care, including DC, before being transferred back to their local hospital for ongoing rehabilitation. In addition, 32% (n=576) of cranioplasty procedures occurred during the same admission as DC, which could be attributed to the fact that patients with head injury often receive their ongoing trauma care and early rehabilitation at major trauma centres and thus remain in a single centre for longer, increasing the options for cranioplasty<12 weeks.

#### Cranioplasty survival

In the survival analysis, the timing of cranioplasty (<12 weeks from DC) had no significant effect on cranioplasty revision rates during the first postoperative year following cranioplasty. This is in line with the literature, which primarily describes conflicting results concerning the effect of the timing of cranioplasty failure and revision rates.^6^ The revision rates in the present 1-year survival analyses mainly comprised those caused by acute and subacute phase complications, such as haematomas and surgical site infections. The long-term issue of bone flap resorption that necessitates cranioplasty revision surgery in 10% of autologous cranioplasty patients^20^ is not fully accounted for, as it mainly occurs in the late phase during the first two postoperative years.^21^

### Limitations

This study had several limitations. First, the study relied on HES data, and coding, especially around DC, can be complex, and potential confounders such as body mass index and coma scores are not captured. In addition, it was impossible to differentiate between primary and secondary DC operations or between smaller craniectomy procedures, for reasons other than decompression. Cranioplasty survival analysis does not consider hydrocephalus, which may be more common at <12 weeks, or late-phase complications, such as bone flap resorption. In addition, early cranioplasty is important in preventing sunken flap syndrome; however, given the limitations of the data, it was not possible to include the impact of this in the results. Patients were censored at the date of death, but we only had this information for in-hospital deaths; therefore, any out-of-hospital deaths were unknown in this study. Although the study demonstrated a lower revision rate for autologous bone than for synthetic implants, no data on the tissue preservation method (subcutaneous implantation or tissue bank storage) were available. However, the impact of this factor on cranioplasty survival could not be assessed.

## Conclusions

The overall number of craniectomies and the subsequent requirements for cranioplasty increased steadily during the study period. However, relating to the timing of cranial reconstruction, there was no evidence of an overall difference in the rate of revision or removal surgery in the early timing group. Having a better understanding of current practice patterns in the UK may aid in cranial reconstruction pathway planning for inpatient and outpatient rehabilitation programmes and aid in dialogue between clinicians, patients and relatives regarding expectations for cranial reconstruction.

## Data Availability

Data are available upon reasonable request. Not applicable.
