# Supplementary material for: Decompressive craniectomy to cranioplasty: a retrospective observational study using Hospital Episode Statistics in England
Source: BMJ Surg Interv Health Technol. 2024 Jun 3;6(1):e000253. doi: 10.1136/bmjsit-2023-000253 (PMC11149159; doi:10.1136/bmjsit-2023-000253)
Supplement: Supplementary data [file bmjsit-2023-000253supp001.pdf]

26/11/2023
Version Final for submission

Appendix

Appendix 1

ICD-10 codes: Cerebrovascular diseases

Code	Description
**I60**	**Subarachnoid haemorrhage**
**I61**	**Intracerebral haemorrhage**
**I62**	**Another nontraumatic intracranial haemorrhage**
**I63**	**Cerebral infarction**
**I64**	**Stroke, not specified as haemorrhage or infarction**
**I65**	**Occlusion and stenosis of precerebral arteries, not resulting in cerebral infarction**
**I66**	**Occlusion and stenosis of cerebral arteries, not resulting in cerebral infarction**
**I67**	**Other cerebrovascular diseases**
**I68**	**Cerebrovascular disorders in diseases classified elsewhere**
**I69**	**Sequelae of cerebrovascular disease**

ICD-10 codes: Head Injury

Code	Description
**S00**	**Superficial injury of head**
**S01**	**Open wound on head**
**S02**	**Fracture of skull and facial bones**
**S03**	**Dislocation, sprain and strain of joints and ligaments of the head**
**S04**	**Injury of cranial nerves**
**S05**	**Injury of eye and orbit**
**S06**	**Intracranial injury**
**S07**	**Crushing injury of head**
**S08**	**Traumatic amputation of part of the head**
**S09**	**Other and unspecified injuries of the head**

Mee H, *et al. BMJ Surg Interv Health Technologies* 2024; 6:e000253. doi: 10.1136/bmjsit-2023-000253

26/11/2023
Version Final for submission

Appendix 2

The Office of Population Censuses and Surveys (OPCS) classification of interventions and procedures (version 4.9) codes used to identify surgical procedures in the present study

OPCS procedure code	Description of Code
V01.1	Cranioplasty using prosthesis
V01.2	Cranioplasty using bone graft
V01.4	Removal of the prosthesis from the cranium
V01.5	Revision of cranioplasty
V01.8	Other specified plastic repair of the cranium
V01.9	Unspecified plastic repair of the cranium
V03.7	Decompressive craniectomy
V03.8	Other specified opening of the cranium
V03.9	Unspecified opening of the cranium
V05.8	Other operations on the cranium, other unspecified
A40.1	Craniotomy evacuation extradural hematoma
A41.1	Evacuation subdural hematoma

Mee H, *et al. BMJ Surg Interv Health Technologies* 2024; 6:e000253. doi: 10.1136/bmjsit-2023-000253
